# Experimental study on the interface characteristics of geogrid-reinforced gravelly soil based on pull-out tests

**DOI:** 10.1038/s41598-024-59297-9

**Published:** 2024-04-15

**Authors:** Jie Liu, Jiadong Pan, Qi Liu, Yan Xu

**Affiliations:** 1 College of Civil Engineering and Architecture, Xinjiang University, Urumqi, 830047 China; 2 Xinjiang Transportation Planning Survey and Design Institute Co. Ltd., Urumqi, 830006 China

**Keywords:** Geogrid reinforcement, Coarse-grained soil, Pull-out tests, Interface parameters, Geogrid–soil interaction, Civil engineering, Petrology

## Abstract

The factors influencing geogrid–soil interface characteristics are critical design parameters in some geotechnical designs. This study describes pull-out tests performed on gravelly soils commonly encountered in the Xinjiang region and reinforced with two types of geogrids. The factors affecting the geogrid–gravelly soil interface properties are investigated with different experimental loading methods (pull-out velocity, normal stress), geogrid types, and soil particle size distributions and water contents. The ultimate pull-out force increases with the normal stress and pull-out velocity. Furthermore, with increasing coarse particle content and water content, the ultimate pull-out force increases and then decreases sharply. Based on these research results, this paper provides reasonable parameters and recommendations for the design and pull-out testing of reinforced soil in engineering structures. In reinforced soil structure design, the grid depth should be increased appropriately, and the coarse particle content of the overlying soil should be between 30 and 40%. During construction, the gravelly soil should be compacted to the maximum compaction at the optimal water content, and the structure should have a reasonable waterproofing system. According to the calculation results of the interface strength parameters, the uniaxial geogrid–gravelly soil interface has a high cohesive force *c*_sg_, which should not be ignored in reinforced soil structure design.

## Introduction

Geosynthetics have been widely used in the past two decades to protect retaining walls, slopes, and embankments. The interaction between the geosynthetic materials and the soil body mainly reflects the reinforcement effect^1^. At the geogrid–soil interface, the resisting shear force mainly arises from the friction between the soil and the surface of the geogrid^2^. The interface characteristics between the soil and reinforcing material, especially the shear strength of the reinforcing soil interface, directly affect the safety and stability of reinforced soil structures. Therefore, the parameters of this interaction must be considered in design calculations^3–5^.

Many scholars have performed experimental research to understand the interface characteristics between the soil and reinforcing materials in reinforced soil. The corresponding tests are mainly interface direct shear and pull-out tests^3,6,7^. Comparing direct shear test and pull-out test results, Xu et al.^8^ found that the direct shear strength τs, interfacial shear strength τds, and pull-out shear strength τp of a geogrid-compacted soil interface were similar. However, since the direct shear test is used to study the interfacial characteristics of reinforced soil, it can reflect only the interfacial strength of the reinforcing material and the soil and not the tensile strength of the reinforcing material or the strength of the whole soil body^9^. Furthermore, the direct shear test cannot fully simulate the double-sided sliding of the reinforcing material and soil and the large deformation characteristics of the soil when it is damaged. However, the pull-out test can consider various factors, such as soil expansion, crowding, and reinforcement slippage, influencing the performance of the reinforcement. This approach can simulate the working conditions of the geogrid inside the soil with simultaneous forces above and below. It can reflect the evolution of the reinforced soil structure during loading^10^. Therefore, the pull-out test is one of the standard methods used to study the reinforcement characteristics of geogrids in soil and to deduce the residual strength and peak strength.

Some domestic and foreign scholars have used this experimental method to investigate the mechanism of reinforcement and the factors influencing the interfacial characteristics of geogrids. Ochiai et al.^11^ conducted field and laboratory pull-out tests to determine the parameters required to design and analyze geogrid reinforcement structures and elucidate their pull-out mechanisms. They noted that geogrids may fracture or elongate under normal stress. In addition, they recommended that pull-out tests be performed at a low normal stress. Li et al.^12^ conducted a series of pull-out tests to investigate and compare the load‒displacement characteristics of tire belts and uniaxial and biaxial geogrid-reinforced sandy soils under different normal stresses. The damage to the tire belt-reinforced sand was progressive, with the shear strength of each part of the sand depending on that of various other parts of the sand. The interlocking effect and pull-out resistance between a tire belt and sandy soil are extreme and significantly greater than those between geogrid and sandy soil. Cardile et al.^13^ investigated the stability of a geosynthetic–soil interface under cyclic loading. Under specific conditions, pull-out resistance parameters should be considered when designing geosynthetic-reinforced soil structures. Derksen et al.^14^ designed a test instrument where the interface between the reinforcing materials and the soil could be observed to study the interfacial interactions occurring along the direction of the reinforcing materials. Three regions were identified based on different interaction patterns. Chen et al.^15^ conducted a comprehensive study of extensive box pull-out tests using the discrete element method. Moreover, large-scale pull-out tests were conducted on embedded biaxial and triaxial geogrid ballast samples. A discrete element model that can reasonably predict the pull-out resistance of geogrid-reinforced soil was developed. Furthermore, the test results indicate that the effect of the geogrid aperture on the tensile strength of the grid is greater than the effect of the geogrid thickness. Perkins and Edens^16^ combined pull-out tests with finite element numerical calculations to establish a numerical finite element model for pull-out tests and simulated pull-out tests with geosynthetic materials. By comparing the results of the finite element analysis and pull-out tests, it was demonstrated that the creep of the geogrid has a slight effect on the deformation of the geosynthetic material. Mosallanezhad et al.^17^ investigated the performance of a new reinforcement system through large-scale pull-out tests and numerical analysis. In the new system, they used cubic units attached to the geogrid with elastic strips. The results showed that the pull-out interaction coefficient of the new system was 100% greater than that of typical geogrid systems. The successful design of geosynthetic reinforcement for geotechnical structures, especially geogrid reinforcement, requires information about the interaction of geogrid–geogrid interfaces. Hajitaheriha et al.^18^ conducted a series of indoor tests and finite element modeling analyses to investigate the significant effects of parameters such as the number of geogrids, burial depth and effective trench depth on the bearing capacity ratio (BCR). The above study suggests that experimental research can not only establish a research model and verify the reasonableness of the model but also provide reasonable design parameters for engineering applications^15^. In addition, the successful design of geosynthetic reinforcement, especially geogrid reinforcement, of geotechnical structures requires information related to the interaction of the reinforcement–soil interfaces.

Factors affecting the characteristics of geogrid–soil interfaces are critical parameters in geotechnical design, so domestic and foreign scholars have studied this topic. Jing et al.^19^ used the discrete element method to simulate the pull-out testing of geogrid-reinforced ballast to demonstrate the effects of particle shape, geogrid size and friction on a ballasted geogrid system. Du et al.^20^ conducted direct shear and pull-out tests on tailings reinforced with geogrids of different grid sizes to explore reasonable grid sizes. The results show that the ratio of the geogrid–tailings interface area to the shear surface area should be controlled between 0.47 and 0.55, within which the embedding and occlusion function of the transverse ribs of the geogrid can be fully exploited so that the reinforcement effect of the geogrid can be optimized. Abdi et al.^21^ designed and developed a sizeable pull-out test apparatus to evaluate the interaction between clay and thin sand layers and geogrids. The effects of factors such as geogrid geometry and soil grain size on pull-out resistance were investigated to facilitate the use of poor-quality soils in engineering. Zhao et al.^22^ investigated the frictional characteristics of biaxial geogrid-reinforced soil at different pull-out velocities and embedment lengths on self-developed test equipment. The test results show that the pull-out velocity has little effect on the shear strength of reinforced soil. However, the pull-out force increases with increasing embedment length. The obtained results are of reference value for the design of biaxial geogrids in engineering. To test the pull-out performance of uniaxial polypropylene geogrids, Baykal and Dadasbilge^23^ conducted pull-out testing to analyze the effect of the geogrid displacement velocity, load magnitude, and specimen width on the specimen behavior. The results show that the boundary effect of the pull-out box affects the peak value of the pull-out test.

An overview of the above research shows that the experimental study of the interface characteristics of reinforced soil is an essential element in the study of the functional properties, damage mode, and reinforcement mechanism of reinforced soil structures, which is of great significance for reducing engineering costs and engineering accidents. The factors influencing the characteristics of the geogrid–gravelly soil interface are critical to understand for predicting the reinforcement–soil interface properties and reinforcement mechanism. There are currently only a few studies on the geogrid–gravelly soil interface characteristics in Xinjiang. This study investigated three categories of gravelly soils in Xinjiang via pull-out tests with different normal stresses, pull-out velocities, and soil water contents. Sandy soil was used to artificially formulate two types of gravelly soil with five gradations each. The influences of the particle shape and gradation of the gravelly soil on the interfacial characteristics of the geogrid were investigated. On this basis, reasonable parameters and suggestions were given for the structural design of the reinforced soil project and pull-out test. The findings of this study will hopefully promote the application of geogrids in gravelly soil roadbeds in Xinjiang.

## Pull-out testing of the geogrid

### Test device

**Figure 1:**
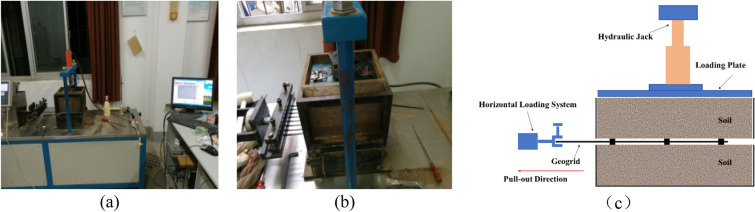
YT140 pull-out tester for geosynthetics: (**a**) test apparatus, (**b**) loading box, (**c**) schematic diagram of the device.

The laboratory instrument used in this study was a YT140 pull-out tester for geosynthetics at the Wuhan University of Technology, College of Transportation (Fig. 1). The YT140 instrument can perform pull-out tests for geogrids, geomembranes, geotextiles, and other geosynthetic materials. The horizontal loading system consists of a displacement sensor and a pull-out force sensor, which can adjust the pull-out force and displacement during the test. A hydraulic device loads the normal stress at constant pressure. The instrument can record the data changes at each stage during the pull-out test in detail.

### Test materials

**Figure 2:**
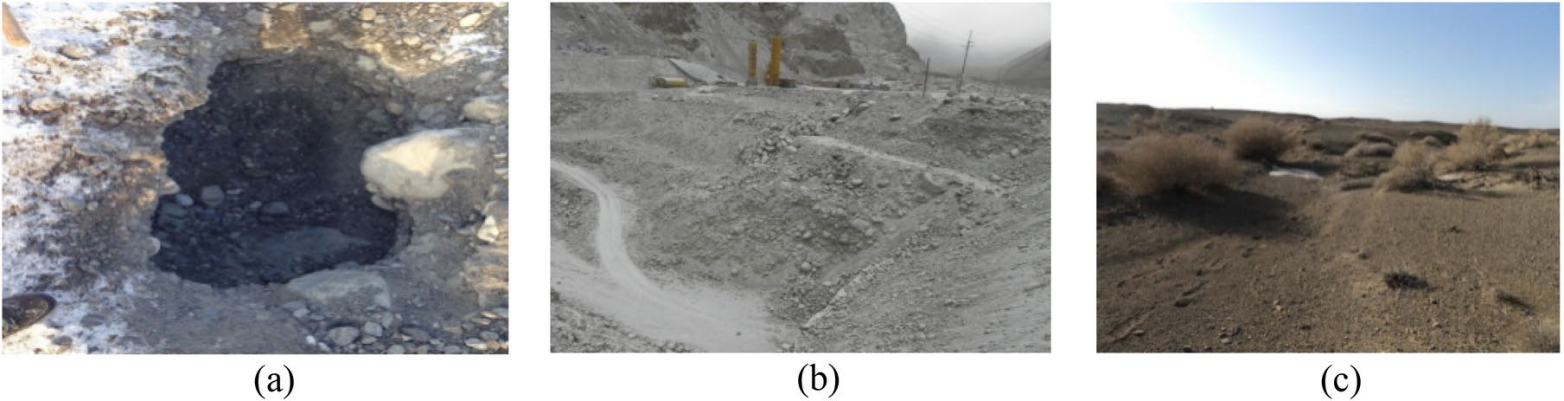
Soil sampling location: (**a**) round gravelly soil, (**b**) angular gravelly soil, (**c**) sandy soil.

The round gravelly soil, angular gravelly soil, and sandy soil studied in this experiment were collected from three different areas in Xinjiang (Fig. 2). The round gravelly soil was from the soil extraction site of the road construction project of the Shawan section of the S101 line in the Tacheng area, Xinjiang. It is a widely used roadbed filler for mountain highways in Xinjiang. The angular gravelly soil was from the soil quarry in Aketao County, Kechu, Xinjiang, and is a poorly graded gravel; it is a typical angular gravelly soil. The sandy soil was from the territory of the Xinjiang Hami region, and the site is located on a piedmont impact plain. The parent rock of this sandy soil is dominated by sandstone and siliceous rock. The three-phase proportion indices of the three gravelly soils derived from compaction testing are shown in Table 1.

**Table 1:** Three-phase proportion indices for the three gravelly soils.

Sample type	Sample no.	Maximum dry density (g/cm^2^)	Optimum water content (%)	Maximum dry density at 92% compaction (g/cm^2^)
Round gravelly soil	S1	2.28	4.6	2.10
Angular gravelly soil	S2	2.25	5.6	2.07
Sandy soil	S3	2.23	6.8	2.05

**Figure 3:**
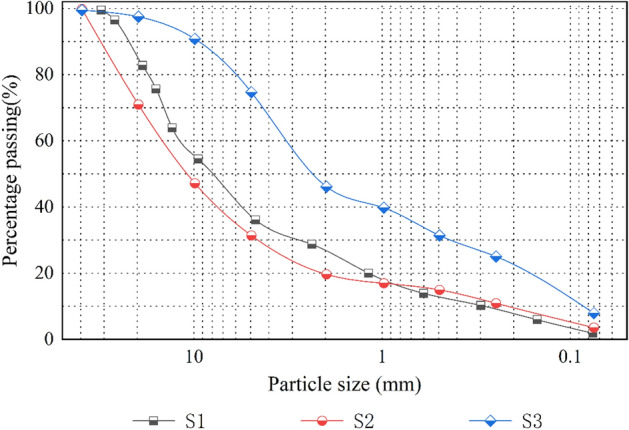
The soil gradation curves.

Due to the limitation of the instrument size, particles greater than 60 mm were removed from the gravelly soils. To maintain the skeletal role of such coarse particles, the continuity of the coarse grain gradation, and a performance similar to that with a natural gradation, the equal mass substitution method was used to convert the content of extralarge particles. That is, all coarse materials are described as a proportion of equal replacement of extralarge particles (to allow the maximum particle size to correspond to the 5 mm particle size content)^24–26^. The scale-reduced gradation curves of the three gravelly soils for the test are shown in Fig. 3.

**Figure 4:**
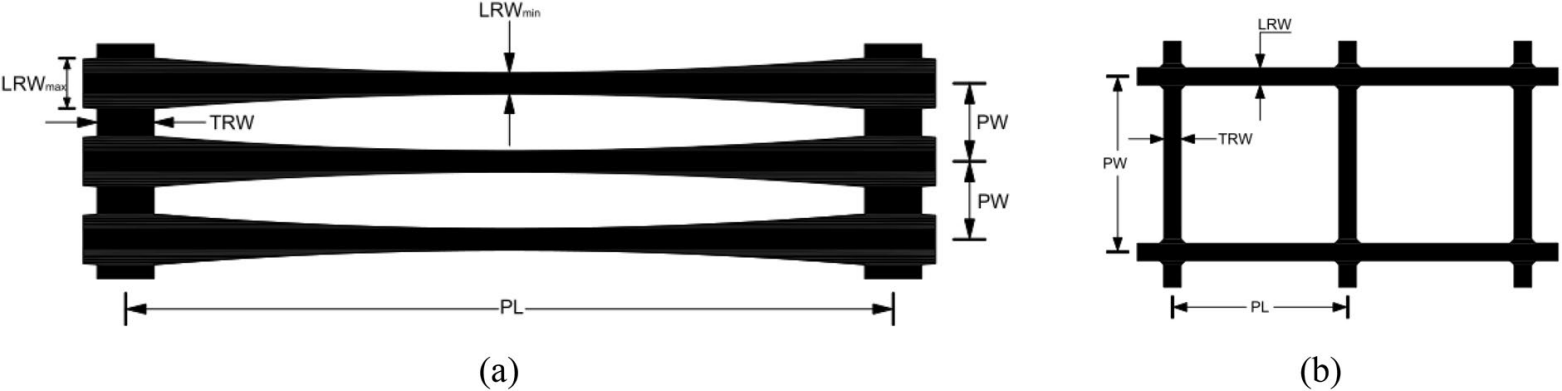
Schematic diagram of geogrids: (**a**) uniaxial geogrids and (**b**) biaxial geogrids.

The uniaxial geogrid used in the test was TGDG50HDPE (Fig. 4), with 46 longitudinal ribs per meter, and the maximum thickness of the horizontal ribs was 1.38 mm. The biaxial geogrid used for the test was a polypropylene biaxially oriented geogrid, model TGSG15-15 (Fig. 4). The geometric and strength characteristics of the geogrids are shown in Table 2.

**Table 2:** Dimensional and physical characteristics of the geogrid.

Characteristics	Direction	TGSG15-15 value	TGDG50HDPE value
Tensile load at 2% strain (kN/m)	Vertical	5.7	72.1
	Horizontal	6.7	
Tensile load at 5% strain (kN/m)	Vertical	11.0	16.3
	Horizontal	11.7	
Nominal elongation (%)	Vertical	27.6	18.0
	Horizontal	19.8	
Maximum tensile load (kN/m)	Vertical	30.7	31.7
	Horizontal	27.0	
Axial stiffness (kN/m)		335	900
Pitch length PL (mm)		35.88	220.0
Pitch width PW (mm)		34.68	17.6
Longitudinal rib width LRW (mm)		3.88	6.5–15.5
Transverse rib width TRW (mm)		5.0	17.5

The geogrid was cut according to the dimensions of the YT140 geosynthetic material pull-out tester loading box. The geogrid specimen used in this pull-out test contained eight vertical ribs along the width direction. The total length was 255 mm, the net length after deducting the distance inside the fixture was 237.5 mm, and the initial width of the geogrid buried in the soil was 100 mm.

### Test design

Before the start of testing, the YT140-type geosynthetic pull-out instrument was calibrated. The standard calibrator fixed in the equipment recorded the measured value during tensile testing, which was compared to the standard value; then, the instrument was adjusted so that the error was within a reasonable range. Following the operation method stipulated in the “Test Methods of Geosynthetics for Highway Engineering” (JTG E50-2006), the geogrid was sampled across 75% of the width of the test box. To ensure that the reinforcing material could not be pulled out of the loading box and that a sufficient anchorage length was reserved, 300 mm was considered along the length direction. First, the lower box was filled in layers and compacted according to the set degree of compaction, and the loose soil particles on the surface were brushed off with a wire brush after each layer of filling to ensure a rough surface and a tight bond between the soil layers, with the top layer of the filling surface initially reaching slightly higher than the lower edge of the seam opening. After the lower box was filled, the specimen was pre-pressurized. The surface was cleaned after the prepressurization treatment so that the fill surface was flush with the lower edge of the seam opening. Then, the buried length of the 10–15 cm geogrid was centered and flatly laid on the soil surface of the lower box. The tensile end of the geogrid aligned with the seam opening between the upper and lower boxes and connected to a horizontally oriented fixture. A plate with a narrow slit of an adjustable height was inserted so that the positive lower edge was on the specimen's surface to fix the plate's position. Subsequently, the filling of the test box was continued in layers, and the layers were compacted until the compacted soil surface was flat and slightly below the top of the box. Finally, the pressurized plate was placed on top, and prepressure was applied for consolidation; the consolidation time was at least 15 min.

After the specimen preparation, a small amount of horizontal load was applied so that the horizontal loading device became taut, and the pull-out force of the instrument was set to zero. The pull-out velocity was set, a horizontal force was applied, pulling started, and after the pull-out force reached the peak, the test continued until it stabilized and then stopped. The pull-out force gradually pulled out the geogrid from the system. If no peak pull-out force occurred or the specimen was pulled out of the box as a whole, the length of the geogrid buried in the soil was shortened, and the test was repeated. The test program is shown in Table 3.

**Table 3:** Test program.

Test condition	Sample no.	Geogrid type	Variant
Condition 1	S1	Uniaxial geogrids	Normal stress
Condition 2	S1/S2	Uniaxial geogrids	Pull-out velocity
Condition 3	S1/S2/S3	Biaxial geogrids	Particle shape and gradation
Condition 4	S3	Uniaxial geogrids	Water content

**Figure 5:**
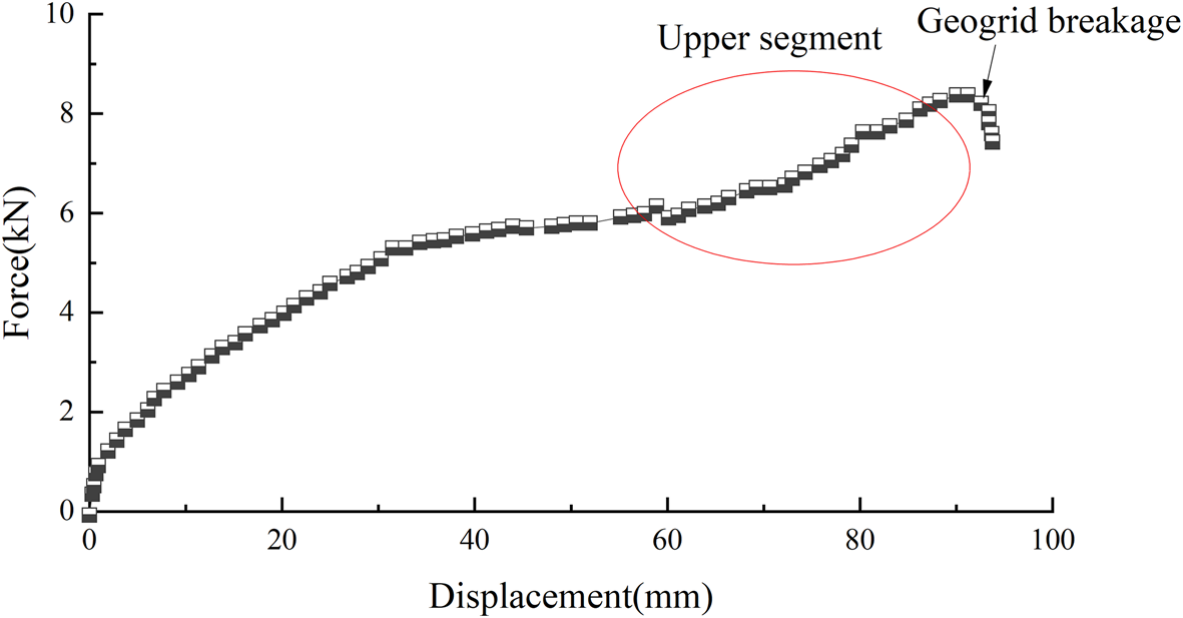
Force‒displacement curves of the S1 soil samples at a normal stress of 100 kPa.

In a pull-out test, the boundary effect of the sidewall of the pull-out box cannot be neglected. Figure 5 shows the pull-out curve of the uniaxial geogrid in the S1 soil sample when the normal stress is 100 kPa. After the pull-out test starts, the curve exhibits an obvious upward trend, after which the pull-out force decreases sharply. This is due to the increase in the pull-out displacement; geogrid mesh holes on the soil body of the embedded fixation effect lead to movement of the soil particles to the pull-out outlet, resulting in an increase in the density of the region near the pull-out outlet until the final geogrid becomes stuck in the pull-out outlet, resulting in a sharp increase in the pull-out force and ultimately in geogrid fracture. In contrast, this situation does not occur in actual projects because there are no fixed sidewall constraints. If the pull-out box is large enough, the geogrid can also break before it is pulled out. Therefore, the elimination of the boundary effect can only be performed by correcting the pull-out force–pull-out displacement curve. If the pull-out curve shows an upward section with a sharp increase in the pull-out force, this section is removed.

## Pull-out test results and analysis

### Analysis of the force‒displacement curves of the pull-out tests under various normal stresses

**Figure 6:**
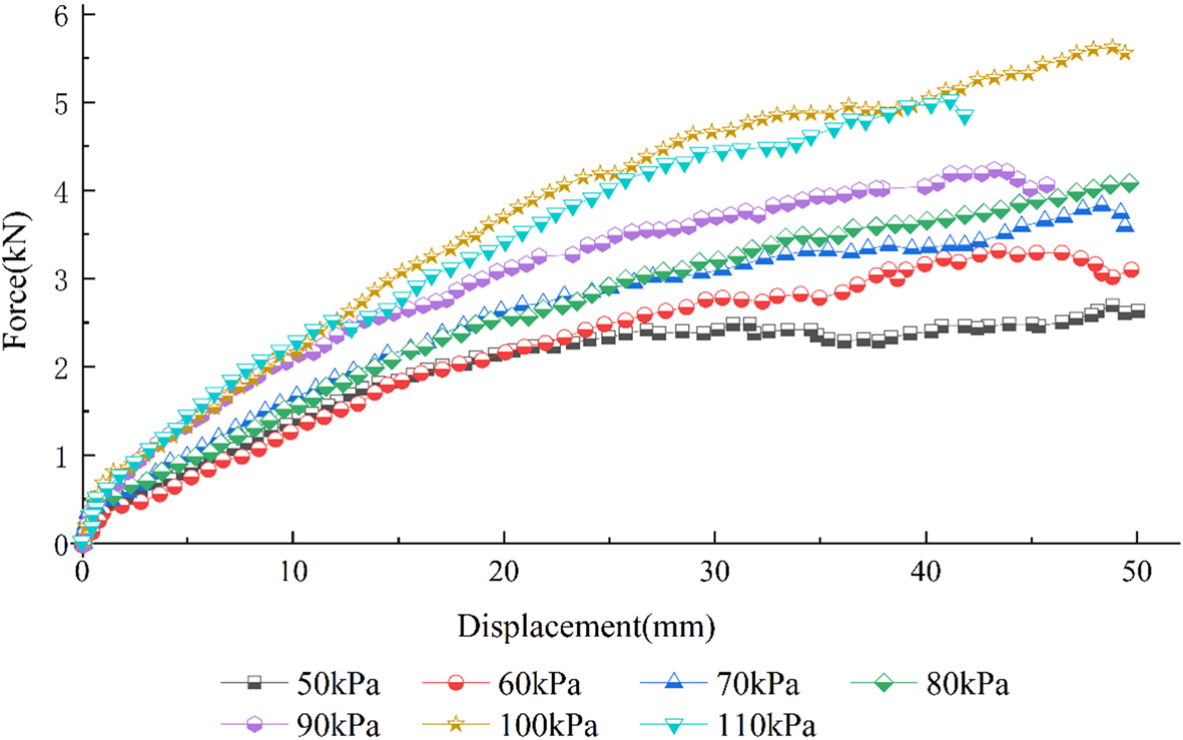
Force‒displacement curves of pull-out tests of S1 soil under various vertical loads.

To study the influence of normal stress on the characteristics of the geogrid–soil interface in the pull-out test, a TGDG50HDPE uniaxial geogrid was used as the reinforcing material, and the S1 soil was used as the filler. A total of 7 groups of geogrid–gravelly soil pull-out tests with different normal stresses were carried out with a pull-out velocity of 1.0 mm/min. As shown in Fig. 6, the curve between the pull-out displacement and force varies widely under different normal stresses. The pull-out force increases with increasing pull-out displacement, and the relationship between the pull-out force and pull-out displacement corresponds to strain hardening. At the beginning of the pull-out tests, the curves of the relationship between the pull-out displacement and force under different loads all have a linear segment for small pull-out displacements. This segment is the static friction stage, and its slope increases with normal stress. The pull-out displacement in this section mainly reflects the deformation of the geogrid. The greater the normal stress is, the longer the static friction stage, and the greater the pull-out force. After the linear static friction stage ends, the curve between the pull-out displacement and force increases linearly. The analysis shows that the curves of the relationship between the pull-out displacement and force under different normal stresses can be separated into two groups at this stage. The slope of the curve under 90–110 kPa of normal stress is significantly larger than that under 50–80 kPa of normal stress, and the pull-out force increases faster with increasing pull-out displacement. Afterward, the curves of the relationship between the pull-out displacement and force enter a nonlinearly increasing phase, in which the pull-out force of the geogrid increases at a slower velocity with increasing pull-out displacement. When the pull-out displacement reaches a certain level, the pull-out force peaks. Finally, the each curve of the relationship between the pull-out displacement and force ends with the peak pull-out force remaining stable or the geogrid fracturing.

The relationship between the normal stress and the peak pull-out force was analyzed by comparing the geogrid force‒displacement curves of the pull-out tests under various vertical loads. When the normal stress is low, the force between the soil particles and the force between the soil and geogrid are small, the movement of the soil particles is easier to achieve, the embedment effect on the horizontal ribs of the geogrid is small, and the friction between the soil and geogrid is also very small. Hence, the pull-out force increases slowly with the pull-out displacement at the late stage of the test. This increase is mainly due to the resistance of the soil particles embedded in the mesh and the horizontal ribs before the crowding of the horizontal ribs^27^. When the normal stress is high, the force transmitted between the soil particles and the soil particles to the geogrid is large, and the friction force is high^28^. The more significant shear dilation effect at the interface between the soil particles and the geogrid makes the soil particles continuously compact. The embedment effect between the soil and the geogrid is more prominent. As a result, the pull-out resistance continues to increase, and the pull-out displacement corresponding to the peak strength increases.

The above analysis shows that the interface strength parameters should differ under the different normal stresses and peak pull-out forces on the geogrid in different layers. At present, the relevant specification does not consider this pattern. Applying a specific vertical load on the upper part of the geogrid or appropriately increasing the overburden thickness can improve the stability of the lower geogrid-reinforced soil.

### Analysis of the force–displacement curves of the pull-out tests under various pull-out velocities

The TGDG50HDPE uniaxial geogrid was used as the reinforcing material, and the S1 soil and S2 soil were used as fillers to conduct pull-out tests at different pull-out velocities under 100 kPa of normal stress to study the effect of the change in pull-out velocity on the mechanical characteristics of the geogrid-reinforced gravelly soil. The pull-out velocity of the geogrid–soil pull-out test can be selected according to the site soil material and drainage conditions, as well as the consolidation rate of the soil samples. According to the “Test Methods of Geosynthetics for Highway Engineering” (JTG E50-2006), the corresponding range for this study is generally 0.2–3.0 mm/min. Thus, four different pull-out velocities of 1.0, 1.5, 2.0, and 3.0 mm/min were considered, and the force‒displacement curves of the pull-out tests were determined under a normal stress of σ = 100 kPa.

**Figure 7:**
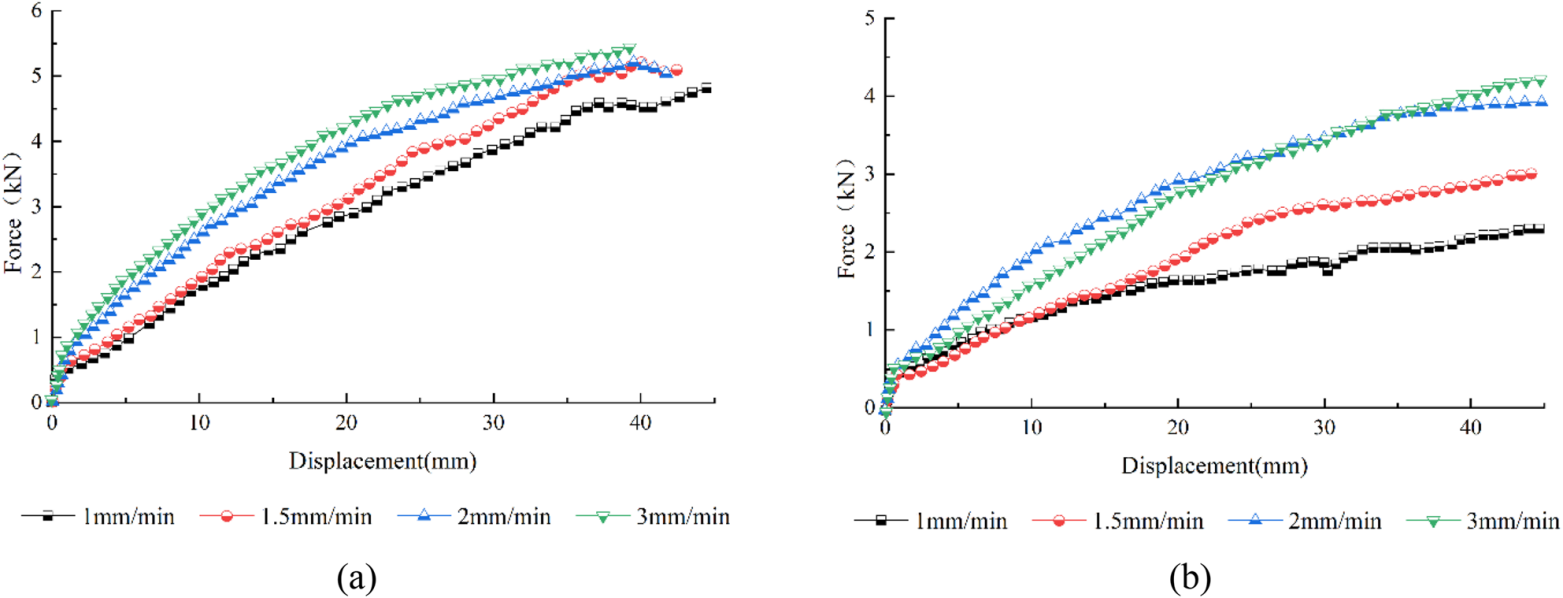
Force‒displacement curves of pull-out tests under various pull-out velocities: (**a**) S1 soil, (**b**) S2 soil.

Figure 7a, b show the geogrid force‒displacement curves of the pull-out tests for the S1 soil and S2 soil at different pull-out velocities. The overall curves of both soil samples exhibit strain hardening for all four pull-out velocities considered.

Figure 7a shows that the greater the pull-out velocity is, the greater the rate of increase in the pull-out force in the middle of the pull-out stage. Moreover, the difference between the peak pull-out force and the corresponding pull-out displacement is relatively small at the tested pull-out velocities. Because the coarse particles of round gravelly soil are spherical, the particles are more likely to move and rotate when subjected to a pull-out force, which is more likely to dissipate the dilatancy effect. Therefore, the round gravelly soil particles will be rearranged after a specific pull-out displacement.

Figure 7b shows that the larger the pull-out velocity, the more pronounced the strain hardening phenomenon of the angular gravelly soil is, the faster the pull-out force increases with the pull-out displacement, and the larger the peak pull-out force is^22^. Compared to the peak pull-out force at a pull-out rate of 1 mm/min, the peak pull-out force at rates of 1.5 mm/min, 2 mm/min, and 3 mm/min increases by 30.7%, 70.6%, and 83.3%, respectively. This means that when the pull-out velocity is small, the relative displacement of the geogrid–gravelly soil interface is small per unit of time, and the geogrid has a long travel time to complete the displacement. The soil particles in the interface range have a stress concentration at the horizontal ribs that dissipates continuously with the rearrangement of soil particles. The stress of the reinforcing material should be evenly distributed, and the required pull-out force should be small. The larger the pull-out velocity is, the larger the relative displacement at the geogrid–soil interface per unit time. Additionally, the soil particles within a specific range above and below the geogrid–soil interface cannot readjust. Thus, the stress concentration at the horizontal ribs cannot dissipate, causing the soil near the geogrid to undergo shear dilation^29^. A significant interfacial frictional resistance is generated, increasing the peak pull-out force as the pull-out velocity increases.

The interaction mechanism between the geogrid and soil is more complex and closely related to the loading rate. In engineering applications, the mechanical performance index parameters should be determined through tests according to the actual conditions of the project. The geogrid–gravelly soil reinforcement structure takes some time to stabilize. Considering the safety of the structure, it is recommended to select a pulling speed of 1.5–2 mm/min for round gravelly soils and 1 mm/min for angular gravelly soils when selecting structural calculation parameters.

### Analysis of the force‒displacement curves of the pull-out tests under various particle shapes and gradations

**Figure 8:**
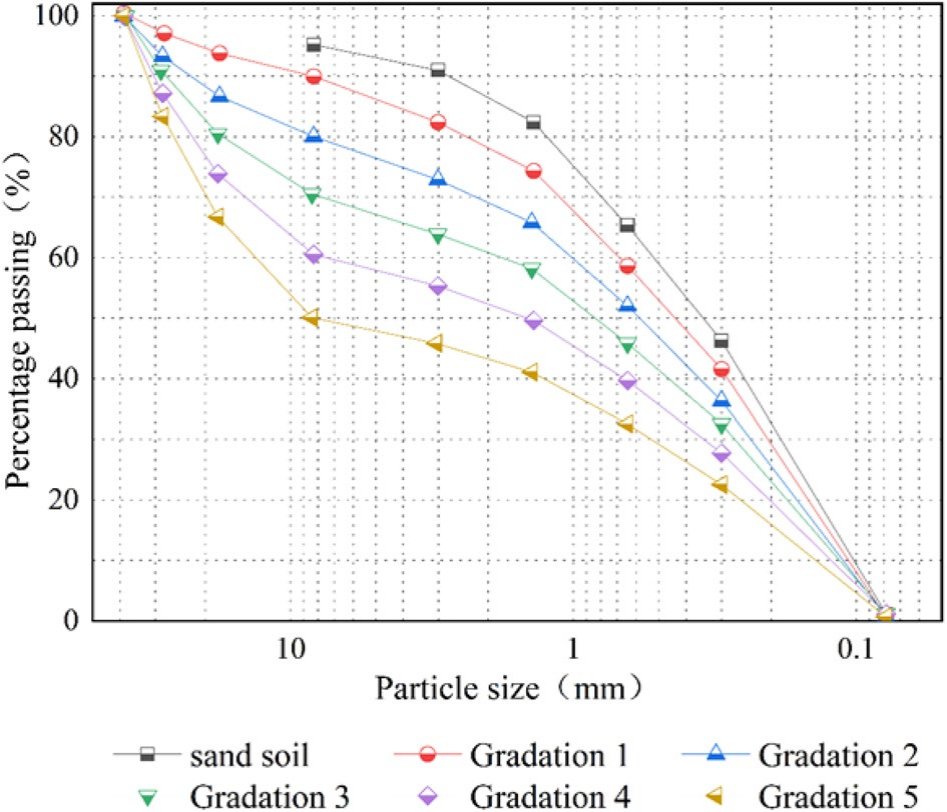
The gradation curves of artificially prepared gravelly soil.

To study the effect of the gravelly soil particle shape and gradation on the geogrid–gravelly soil interface characteristics, coarse particles larger than 5 mm were sieved out of the S3 sandy soil, and the remaining fine particles were retained as the fine particle fraction of the test soil material. Compaction tests were performed on the fraction of fine particles less than 5 mm, yielding a maximum dry density of 1.61 g/cm^3^ and an optimum water content of 6.1% for the fine particles. Crushed stone and pebble stone of 1 to 2 cm were used as the coarse grains of the gravelly soils and mixed with fine-grained soils in different proportions to make five gradations ranging from fine to coarse and a total of ten different gradations of angular gravelly and round gravelly soils. The compaction of these ten soil gradations was converted using the maximum dry density and optimum water content of the fine-grained fraction less than 5 mm to ensure that the compaction remained consistent. The grading scheme and physical properties are shown in Table 4. The gradation curves of the five artificially formulated gravelly soils and the gradation curves of the sandy soil are shown in Fig. 8.

**Table 4:** List of grading schemes and parameters.

Grading scheme	Round gravelly soil		Angular gravelly soil		*C*_*u*_	*C*_*s*_	Packing density (g/cm^3^)
	Sandy soil (%)	1– 2 cm round gravelly soil (%)	Sandy soil (%)	1 – 2 cm angular gravelly soil (%)			
Sandy soil	100		100		3.96	0.79	1.61
Gradation 1	90	10	90	10	4.75	0.65	1.68
Gradation 2	80	20	80	20	6.57	0.50	1.75
Gradation 3	70	30	70	30	10.96	0.32	1.82
Gradation 4	60	40	60	40	36.79	0.10	1.91
Gradation 5	50	50	50	50	53.73	0.08	2.00

**Figure 9:**
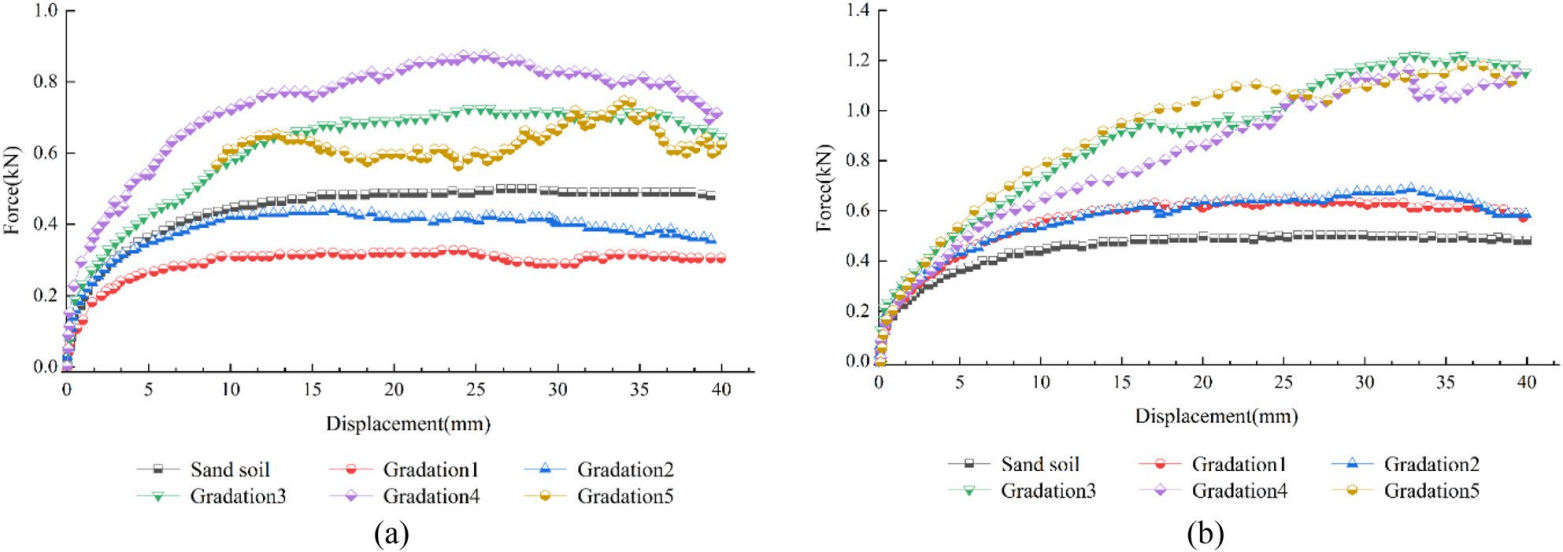
Force‒displacement curves of pull-out tests: (**a**) round gravelly soil, (**b**) angular gravelly soil.

A TGSG15-15 biaxial geogrid was used as the reinforcing material for these tests. The force‒displacement curves of the pull-out tests of round gravelly soil and angular gravelly soil with different gradations were obtained under a 50 kPa normal stress and a 2.0 mm/min pull-out velocity, as shown in Fig. 9a, b.

Figure 9a shows that the peak pull-out force of the sandy soil (0.51 kN) at a normal stress of 50 kPa is greater than the peak pull-out force of gradation 1 (0.44 kN) and gradation 2 (0.31 kN) with a lower content of round gravel. Because the surfaces of the pebble-like coarse particles used in this test are smooth, the friction coefficient is lower than that of the sandy soil, which reduces the internal friction angle φ of the mix. Therefore, the coarse particles distributed in the sandy soil are separated from each other when the coarse particle content is low, it is difficult to achieve mutual occlusion, and the pull-out force is smaller than that of pure sandy soil. The peak strength of gradation four among the five round gravelly soils is the largest at 0.88 kN, 1.73 times that of the sandy soil, 2.0 times that of gradation 1, and 2.73 times that of gradation 2. The difference in peak strength between gradation 3 and gradation 5 is minor, and the peak strengths are 0.73 kN and 0.66 kN, respectively.

Figure 9b shows that the peak pull-out forces of the angular gravelly soil at a normal stress of 50 kPa are greater than the peak pull-out forces of the sandy soil. Among the five angular gravelly soils, the peak pull-out force of gradation 3 is 1.22 kN, which is 2.41 times that of the sandy soil, 1.91 times that of gradation 1, and 1.77 times that of gradation 2. The difference in the peak pull-out forces between gradation 4, 1.17 kN, and gradation 5, 1.11 kN, is small.

Figure 9a, b show that the sandy soils of gradations 1 and 2 have similar trends for the curve segments after the peak pull-out force. Round gravelly soil with a higher coarse particle content (gradation 3 to gradation 5) exhibits strain softening. Gradation 3 to gradation 5, with higher coarse-grained contents, of angular gravelly soil show strain hardening characteristics in the curve's rising section after the pull-out force reaches its peak value. For gradation 3 to gradation 5, the angular gravelly soil and round gravelly soil, the pull-out forces developed faster and peaked earlier than those of the other three groups of tests. This indicates that the coarse grains are involved in the embedded fixation effect earlier and that only a very small pull-out displacement is required to achieve a specific strength. Meanwhile, comparing the force‒displacement curves of the pull-out tests of the angular gravel and round gravelly soils, it can be seen that the more coarse-grained material there is, the more pronounced the curve fluctuation, showing a clear step-like shape. When more coarse particles are present, the rotation, locking, and movement of soil particles affect the pull-out force of the geogrid more. In addition, with 1–2 cm coarse grains, the gravel material gradation is not uniform, and the large particle distribution dramatically influences the curve during the pull-out test. The large particles at the nodes and horizontal ribs of the geogrid are pushed as the geogrid is pulled. The adjustment of the misaligned large particles in the mesh increases the resistance of the large particles after realignment. Thus, the fluctuation in the force‒displacement curve of the pull-out test is more pronounced, showing an apparent step-like shape^30^.

**Figure 10:**
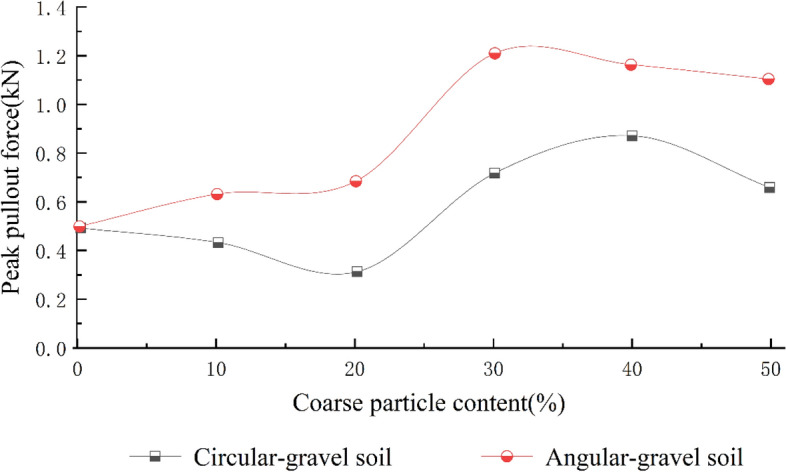
Curves of the peak shear stress of specimens with coarse particles.

Figure 10 shows that when the content of particles larger than 5 mm in the test material is 30%, the pull-out friction effect is substantially greater than that of general sandy soils. When the gravel content is between 30 and 40%, the fine particles in the gravelly soil fill the pores between the coarse particles, making the material denser. The responses of the coarse and fine particles in this case are coupled, and the contact area with the geogrid surface will reach a maximum. Conversely, when the gravel content exceeds 40%, the large particles play a skeletal role, and the fines are too small to fill the pores between the large particles. The frictional effect between the soil and reinforcement is then reduced^31^. This shows that appropriately increasing the content of coarse particles in gravelly soils can improve the shear strength of the reinforcement–soil contact surface and its residual shear strength^32^.

The peak strength of the coarse grains of the irregular angular gravelly soil is generally significantly greater than that of the round gravelly soil with rounded coarse grains under the same working conditions in the pull-out test, indicating that the reinforcement effect of angular gravelly soil is greater than that of the round gravelly soil. Generally, in pull-out tests, angular gravelly soil has a peak strength that is 30% to 40% greater than that of round gravelly soil of the same gradation.

The above analysis shows that when the content of coarse particles with particle sizes greater than or equal to 1 cm is greater than 30%, the peak strength of the pull-out test is the greatest observed in this work. To ensure a good reinforcement effect, the content of coarse particles in geogrid-reinforced gravelly soil is recommended to be 30% to 40% for structure design, and it is recommended that gravelly soil with angular particles is used as roadbed filler.

### Analysis of the force‒displacement curves of the pull-out tests under various water contents

In engineering practice, rainfall is the cause of damage to reinforced structures, and the water content is the main factor affecting the stability of reinforced structures^33^. To study the influence of the water content in the reinforced soil on the geogrid–soil interface characteristics, a pull-out test of the S3 soil sample under a 50 kPa normal stress and a 2.0 mm/min pull-out velocity was carried out by using a YT140 pull-out tester for geosynthetics with TGDG50 uniaxial geogrids under six groups of different water contents. The ultimate pull-out force of the geogrid was tested for different water contents.

**Figure 11:**
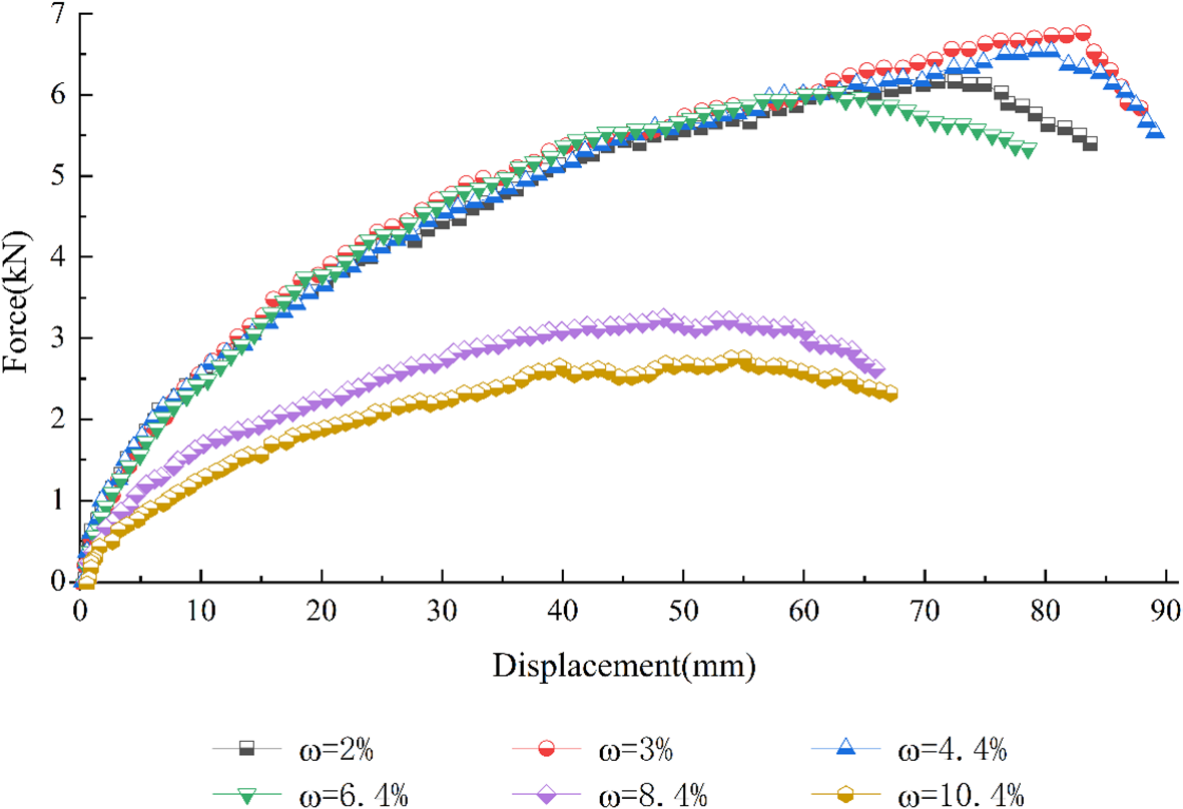
Force‒displacement curves of the pull-out tests of S3 soil with different water contents.

Figure 11 shows the force‒displacement curves of sandy soils with different water contents from the pull-out tests. A clear differentiation in the curve shapes occurs at a water content of 6.4%. When the water content ranges from 2% to 6.4%, the force‒displacement curves of the pull-out test overlap at the beginning of the pull-out displacement, and the pull-out force increases faster with increasing displacement. When the water content ranges from 2 to 6.4%, the higher the water content, the earlier the peak pull-out force appears. The force‒displacement curves of the pull-out test show strain softening after the peak value, and the curve has a decreasing trend. When the water content is more significant than 6.4%, the peak pull-out force is lower, and the pull-out force increases more slowly with increasing pull-out displacement. The force‒displacement curves of the pull-out tests show a peak followed by a flat section, reflecting strain hardening. The displacement required to reach a specific pull-out force is greater at a higher water content.

**Figure 12:**
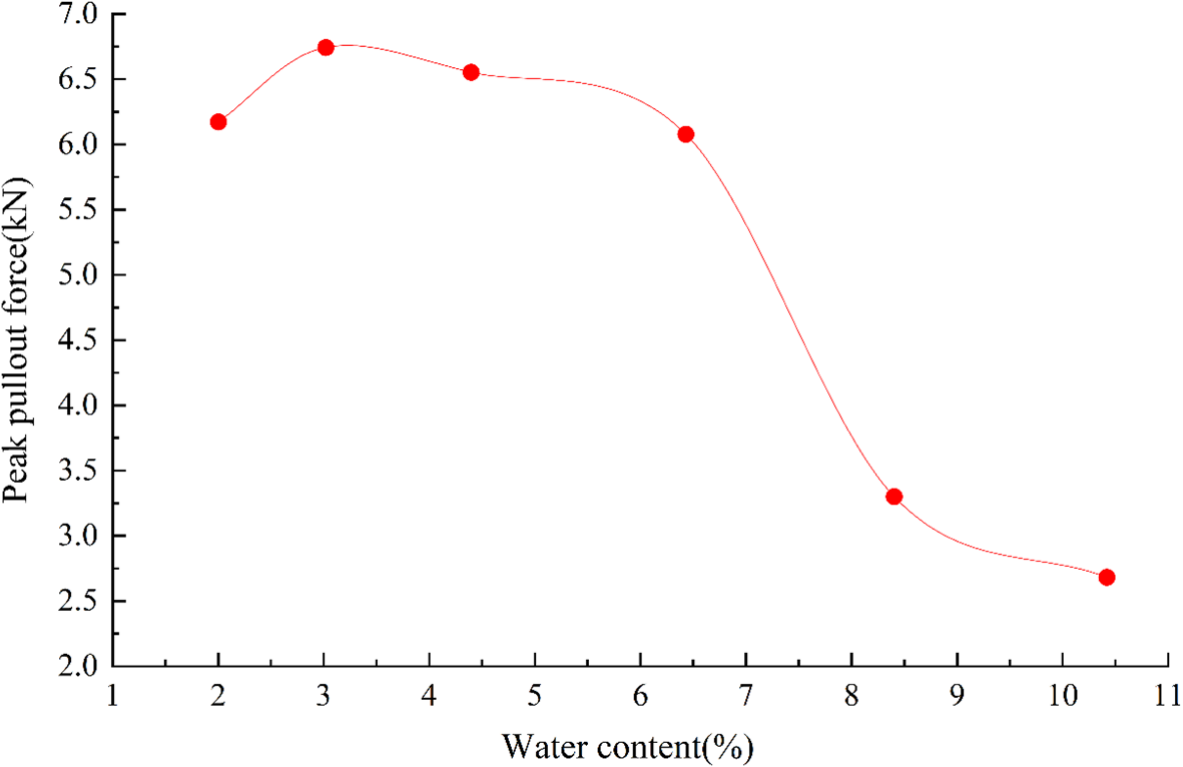
Relationship between the ultimate pull-out force and water content of gravelly soil.

Figure 12 shows the relationship between the ultimate pull-out force and the water content of gravelly soil. Clearly, the ultimate pull-out force increases and then decreases with increasing water content. The role of geogrids in reinforcing gravelly soil is mainly related to friction and embedded fixation. At low water contents, pseudocohesion occurs in gravelly soils due to capillary water action at the edges. The pseudocohesion will initially increase with increasing water content, and the resistance of the surrounding soil particles to movement when the geogrid is pulled increases. When the pseudocohesion reaches its maximum value, the pull-out resistance also peaks. Subsequently, the pseudocohesion decreases as the water content increases until it disappears, and the pull-out resistance decreases. When the pseudocohesion disappears, the water content then increases. At this point, the water acts as a lubricating fluid between the soil particles and at the contact surface between the geogrid and the soil particles. The higher the water content is, the more pronounced the lubrication effect will be such that the pull-out resistance will decrease sharply with increasing water content.

In addition, the sandy soil used in this test is a typical cohesionless soil. When the water content in the soil changes, the friction coefficient between the soil particles and between the geogrid and soil particles decreases with increasing water content, which leads to a decrease in the friction between the soil and grids. Moreover, when the water content approaches the optimum water content, the compaction of the fill gradually increases, and the embedment effect of soil particles on the mesh becomes more pronounced^34^. The resistance of the transverse rib to the soil particles gradually dominates. As the water content continues to increase, the friction between the soil and the geogrid continues to decrease, the compaction of the soil decreases, the resistance of the horizontal rib to the soil particles gradually decreases, and the ultimate pull-out force appears to decay sharply^35^.

According to the above analysis, during the construction of reinforced structures, attention should be given to selecting the two indicators: the water content and compaction degree of the fill soil. A suitable water content should be selected during the construction process and should be at most the optimum water content. The compaction degree should be as close as possible to the maximum for the fill material used. Reinforced structures should be designed to prevent rainwater immersion during the heavy rainfall flood season, as these processes reduce the strength of the structure and lead to structural instability.

## Discussion

On the basis of the above research results, the parameters of the likely cohesion (*c*_sg_) and likely interface friction angle (*φ*_sg_) are introduced for describing the strength of the geogrid–soil interface. For a given soil sample and a given grating, *c*_sg_ and *φ*_sg_ are constants, so *c*_sg_ and *φ*_sg_ are the recommended parameters for use in engineering design and testing. After determining the ultimate pull-out force under different normal stresses based on the pull-out curves of the uniaxial geogrids in three soil samples, the corresponding interface shear strength* τ*_f_ is calculated from Eq. (1). On this basis, the interface strength indices *c*_sg_ and *φ*_sg_ can be obtained by plotting the *τ*_f_–*σ* relationship (Table 5).

1 $${\tau }_{f}{=}\text{0.5} \times \frac{{\text{T}}_{\text{d}}}{{L}_{2}{\text{B}}}$$

where T_d_ is the ultimate pull-out force (kN) and L_2_ is the length of the part of the geogrid buried in the soil (m). B is the width of the geogrid specimen (m).

**Table 5:** Indices of the interface resistance between the uniaxial geogrid and gravelly soils.

Sample no.	*w* (%)	*c*_sg_ (kPa)	*φ*_sg_ (°)	*τ*_f_ (kPa)			
				*σ* (kPa)			
				25	50	100	150
S1	5.6	23.0	40.0	43.7	64.5	106	147.5
	7.6	12.9	38.8	33	53.1	93.3	133.5
S2	4.6	28.1	40.1	45.2	71.5	111.9	153.4
	6.6	32.1	30.6	46.8	61.6	91.2	120.7
S3	6.4	115.3	47.2	142.2	169.2	223.2	277.1
	8.4	87.8	43.1	111.2	134.6	181.4	228.2

Table 5 shows that the interfacial friction angle is greater than 38° in all cases except for the case of S2 soil with a water content of 6.6%. The interfacial friction angle is not less than 40° at the optimum water content. The interface strength between the gravelly soil and the uniaxial grid is very high. Therefore, the water content at the time of rolling during roadbed construction is generally equal to or close to the optimum water content. In addition, the interfacial cohesion between uniaxial grids and gravelly soils is not equal to zero; instead, the interfacial cohesion is more than 10 kPa. Additionally, the *c*_sg_ for S3 soil is even greater than 100 kPa (w = 6.4%) while the current specification^36^ is ignored, which is on the conservative side. Because the cohesive force of the geogrid– gravelly soil interface is not between the two adhesive forces, the cohesive force *c*_sg_ actually reflects the embedded fixation of the geogrid mesh and soil particles, particularly, that of the coarse particles in the gravelly soil and the geogrid holes and cross-ribs; this resistance is considerable, so ignoring it is too conservative.

This study used pull-out tests to study how four factors, namely, the normal stress, pull-out rate, particle shape and gradation, and water content, affect the geogrid–gravelly soil interface properties. According to the research results, reasonable parameters and suggestions are given for future engineering structure design and pull-out testing. In addition, in the design and construction of roadbeds, the type of geogrid should be selected based on the actual force and deformation of the roadbed. Uniaxial geogrids are suitable for resisting unidirectional forces, such as the reinforcement of high-fill roadbeds. Biaxial geogrids are suitable for resisting uneven settlement and deformation in weak roadbeds. However, this study investigated only the force‒displacement curves and peak pull-out forces of geogrid-reinforced coarse-grained soils under the above four factors, and the sample size was small. In the experimental design, not all three soil types were considered in the pull-out tests for each factor due to the limitations of the test conditions. For example, only the S1 soil was adopted in the tests that considered the effect of normal stress on the interfacial characteristics of the reinforced soil, and the S3 soil was not considered in the pull-out velocity tests. However, the patterns derived from these tests can be applied to other soils^3,37,38^. In the future, we will further expand our research on the factors influencing reinforced soils. Moreover, at the microscale, studies on the movement of gravelly soil particles under the action of different factors and changes in the influence zone of reinforcement have yet to be conducted. Using digital image correlation (DIC) or particle image velocimetry (PIV) to study the microscale motions at the reinforcement–soil interface allows for a better analysis of the evolution and distribution of the particle displacement field in the reinforcement influence zone of the soil^39^. At present, effective methods for studying the interfacial characteristics of reinforced soils include pull-out tests and direct shear tests. However, the two methods produce different test data, failure modes, and strength indices in practical tests^40,41^. This study used only the pull-out test to analyze geogrid–gravelly soil interface characteristics. In the future, the results of pull-out test under the same experimental conditions should be compared with the results of direct shear to explore the differences between and advantages and disadvantages of the two tests for studying the interfacial characteristics of reinforced soil.

## Conclusion

In this study, a series of pull-out tests were conducted on geogrid-reinforced gravelly soils to determine the effects of different normal stresses, pull-out rates, soil particle shapes and gradations, and moisture content conditions on the interfacial properties of reinforced soils. Based on the pull-out test data, the interfacial strength parameters of the three types of soils reinforced by uniaxial geogrids were obtained for different normal stresses and water contents. Reasonable parameters and suggestions were given for the structural design of reinforced soil engineering and pull-out testing. The following conclusions were drawn:

1. The pull-out force increases with the pull-out displacement at each of the normal stresses tested, and the ultimate pull-out force increases continuously with the normal stress. Therefore, in the design and construction stage of reinforced soil structures, appropriately increasing the geogrid burial depth is helpful for improving the stability of the geogrid-reinforced soil.
2. The greater the pull-out velocity is, the more pronounced the strain-hardening behavior reflected in the force‒displacement curves of the pull-out tests. The faster the pull-out force continues to increase, the greater the peak pull-out force. Considering the safety of a structure, when choosing the structural calculation parameters, it is recommended to use a pull-out velocity of 1.5 –2 mm/min for the pull-out testing of geogrid-reinforced round gravelly soil and a pull-out velocity of 1 mm/min for the pull-out testing of angular gravelly soil.
3. Among the conditions tested, when the content of coarse particles with particle sizes greater than or equal to 1 cm is greater than 30%, the peak force of the pull-out test is the largest. To ensure a good reinforcement effect, the content of coarse particles in the geogrid–gravelly soil reinforcement structure design is recommended to be 30% to 40%, and it is recommended to prioritize gravelly soil with angular particles as roadbed filler.
4. When the water content in the sandy soil is less than the optimum, the trend of the force‒displacement curves and the peak pull-out forces of the pull-out tests are less different than when the water content is greater than the optimum. However, when the water content exceeds the optimum, the peak pull-out force decreases sharply. Therefore, in the design and construction of geogrid-reinforced soil engineering, special attention should be given to selecting and implementing the two indicators of the fill soil: the water content and compaction of the fill soil should be approximately the optimal water content and maximum compaction. During the construction and operation of geogrid-reinforced soil engineering structures, special attention should be given to the drainage system of the structure to avoid structural failure due to an excessive water content of the fill.
5. Uniaxial geogrid and gravelly soil interface cohesion *c*_sg_ is larger; it is the grille cross rib end bearing resistance embodiment, is not a geogrid—soil interface viscous size of the reflection of the role of the actual engineering design to ignore the role of the *c*_sg_ is unreasonable conservative practice.

## Data Availability

The datasets used and/or analyzed during the current study are available from the corresponding author upon reasonable request.

## References

[1] Wang J, Liu F, Wang P, Cai Y (2016). Particle size effects on coarse soil-geogrid interface response in cyclic and post-cyclic direct shear tests. Geotext. Geomembr..

[2] Liu C-N, Ho Y-H, Huang J-W (2009). Large scale direct shear tests of soil/PET-yarn geogrid interfaces. Geotext. Geomembr..

[3] Kayadelen C, Önal TÖ, Altay G (2018). Experimental study on pull-out response of geogrid embedded in sand. Measurement.

[4] Moraci N (2014). Soil geosynthetic interaction: Design parameters from experimental and theoretical analysis. Transport. Infrastruct. Geotechnol..

[5] Karnamprabhakara BK, Chennarapu H, Balunaini U (2023). Modified axial pullout resistance factors of geostrip and metal strip reinforcements in sand considering transverse pull effects. Geotech. Geol. Eng..

[6] Makkar FM, Chandrakaran S, Sankar N (2019). Performance of 3-D geogrid-reinforced sand under direct shear mode. Int. J. Geotech. Eng..

[7] Makkar FM, Chandrakaran S, Sankar N (2019). Experimental investigation of response of different granular soil-3D geogrid interfaces using large-scale direct shear tests. J. Mater. Civ. Eng..

[8] Xu Y, Williams DJ, Serati M (2018). Measurement of shear strength and interface parameters by multi-stage large-scale direct/interface shear and pull-out tests. Meas. Sci. Technol..

[9] Hai-long Z, Yi-chuan X, Ai-jun Z, Shao-hong Z (2013). Experimental investigation on shear strength of reinforced coarse-grained soil. J. China Inst. Water Resour. Hydropower Res..

[10] Wen-bai L, Jian Z (2009). Experimental research on interface friction of geogrids and soil. Rock Soil Mech..

[11] Ochiai H, Otani J, Hayashic S, Hirai T (1996). The pull-out resistance of geogrids in reinforced soil. Geotextiles Geomembr..

[12] Li LH, Chen YJ, Ferreira PMV, Liu Y, Xiao HL (2017). Experimental investigations on the pull-out behavior of tire strips reinforced sands. Materials (Basel).

[13] Cardile G, Pisano M, Moraci N (2019). The influence of a cyclic loading history on soil-geogrid interaction under pullout condition. Geotextiles Geomembr..

[14] Derksen J, Ziegler M, Fuentes R (2021). Geogrid–soil interaction: A new conceptual model and testing apparatus. Geotextiles Geomembr..

[15] Chen C, McDowell GR, Thom N (2013). A study of geogrid-reinforced ballast using laboratory pull-out tests and discrete element modelling. Geomech. Geoeng..

[16] Perkins S, Edens M (2003). Finite element modeling of a geosynthetic pullout test. Geotech. Geol. Eng..

[17] Mosallanezhad M, Taghavi SHS, Hataf N, Alfaro MC (2016). Experimental and numerical studies of the performance of the new reinforcement system under pull-out conditions. Geotextiles Geomembr..

[18] Hajitaheriha MM, Akbarimehr D, Hasani Motlagh A, Damerchilou H (2021). Bearing capacity improvement of shallow foundations using a trench filled with granular materials and reinforced with geogrids. Arab. J. Geosci..

[19] Jing GQ, Luo XH, Wang ZJ (2014). Micro-analysis ballast-geogrid pull out tests interaction. Appl. Mech. Mater..

[20] Du C, Niu B, Wang L, Yi F, Liang L (2022). Experimental study of reasonable mesh size of geogrid reinforced tailings. Sci. Rep..

[21] Abdi M, Zandieh A, Mirzaeifar H, Arjomand M (2021). Influence of geogrid type and coarse grain size on pull out behaviour of clays reinforced with geogrids embedded in thin granular layers. Eur. J. Environ. Civ. Eng..

[22] Zhao, Y., Yang, G., Wang, Z. & Liang, X. Optimal configuration for the wind-solar complementary energy storage capacity based on improved harmony search algorithm *J. Phys. Conf. Ser.* **2598**, 012016 (2023) (IOP Publishing).

[23] Baykal, G. & Dadasbilge, O. *Geosynthetics in Civil and Environmental Engineering: Geosynthetics Asia 2008 Proceedings of the 4th Asian Regional Conference on Geosynthetics in Shanghai, China.* 174–178 (Springer, 2008).

[24] Hai-tao L, Xiao-hui C (2009). Discrete element analysis for size effects of coarse-grained soils. Rock Soil Mech..

[25] Tang K, Xie X, Yang L (2014). Research on mechanical characteristics of gravel soil based on large-scale triaxial tests. Chin. J. Under Sp. Eng..

[26] Yong-zhen Z, Wei Z, Jia-jun P, Na Z (2015). Effects of gradation scale method on maximum dry density of coarse-grained soil. Rock Soil Mech..

[27] Ismail M, Joohari M, Habulat A, Azizan F (2021). Pull-out resistance of sand-geosynthetics reinforcement. Int. J. Integr. Eng..

[28] Abdi M, Zandieh A (2014). Experimental and numerical analysis of large scale pull out tests conducted on clays reinforced with geogrids encapsulated with coarse material. Geotextiles Geomembr..

[29] Liu J, Wang B, Sun Y (2022). Mechanism and mesoscopic characteristics of indirectly reinforced gravelly soil by a geogrid. Adv. Mater. Sci. Eng..

[30] Guangqing Y, Guangxin L, Baojian Z (2006). Experimental studies on interface friction characteristics of geogrids. Chin. J. Geotech. Eng..

[31] Shengyou, L. *Theory and Technology of Modern Reinforced Soil*. 75–81 (China Communication Press, 2006).

[32] Jia-Quan, W., Biao, W. U., Hong, Z., Qi, Z. & Gang, K. E. Large direct shear test research of interface interaction characteristics of geogrid and coarse grained soil. *J. Guangxi Univ. Sci. Technol. * (2015).

[33] Feng X, Yang Q, Li S, Luan M (2009). Influence of water content on pullout behavior of geogrid in red clay. Chin. J. Rock Mech. Eng..

[34] Altay G, Kayadelen C, Taşkıran T, Kaya YZ (2019). A laboratory study on pull-out resistance of geogrid in clay soil. Measurement.

[35] Chen J-N, Ren X, Xu H, Zhang C, Xia L (2022). Effects of grain size and moisture content on the strength of geogrid-reinforced sand in direct shear mode. Int. J. Geomech..

[36] Giroud, J. P. From geotextiles to geosynthetics: A revolution in geotechnical engineering. Proc. 3rd Int. Conf. Geotextiles, Vienna, 1–18 (1986).

[37] Cai X (2022). Study on interface interaction between uniaxial geogrid reinforcement and soil based on tensile and pull-out tests. Sustainability..

[38] Zhao Y, Yang G, Wang Z, Yuan S (2022). Research on the effect of particle size on the interface friction between geogrid reinforcement and soil. Sustainability.

[39] Liu J (2023). Experimental study on the fine-scale characteristics of a geogrid-gravelly soil reinforcement influence zone. Front. Earth Sci..

[40] Wang Z, Jacobs F, Ziegler M (2016). Experimental and DEM investigation of geogrid–soil interaction under pullout loads. Geotextiles Geomembr..

[41] Kim D, Ha S (2014). Effects of particle size on the shear behavior of coarse grained soils reinforced with geogrid. Materials (Basel).

